# Acute generalized exanthematous pustulosis caused by hydroxychloroquine in a patient with rheumatoid arthritis and *CARD14* mutation: Case report

**DOI:** 10.1097/MD.0000000000036168

**Published:** 2023-11-24

**Authors:** Feng Luo, Xue-Mei Yuan, Hong Xiong, Chang-Ming Chen, Wu-Kai Ma, Xue-Ming Yao

**Affiliations:** a Guizhou University of Traditional Chinese Medicine, Guiyang, China; b The Second Affiliated Hospital of Guizhou University of Traditional Chinese Medicine, Guiyang, China.

**Keywords:** acute generalized exanthematous pustulosis, CARD14, histopathology, hydroxychloroquine, rheumatoid arthritis

## Abstract

**Rationale:**

Acute generalized exanthematous pustulosis (AGEP) is a serious adverse skin reaction characterized by the rapid appearance of densely distributed, small, sterile pustules with erythema. However, its pathogenesis is not fully understood. Hydroxychloroquine is widely used for the treatment of autoimmune diseases. Some patients presenting with AGEP have *IL36RN* and *CARD14* gene mutations. Our report describes a patient with rheumatoid arthritis and AGEP associated with hydroxychloroquine and a newly discovered *CARD14* gene mutation.

**Patient concerns:**

A 28-year-old woman with rheumatoid arthritis, treated with leflunomide therapy without marked relief of joint pain, developed multiple rashes with pruritis covering the body 5 days after switching to hydroxychloroquine treatment.

**Diagnoses:**

Based on the patient’s history, symptoms, and histopathological findings, AGEP was diagnosed.

**Interventions:**

Whole-exome sequencing and Sanger validation revealed no mutations in the *IL36RN* gene; however, a *CARD14* gene mutation was present. The patient was treated using ketotifen fumarate tablets, dexamethasone sodium phosphate, calcium gluconate injection, methylprednisolone injection, vitamins C and B12, hydrocortisone butyrate cream, Reed acne cream, potassium chloride tablets, and pantoprazole enteric-coated capsules.

**Outcomes:**

The rash improved after 15 days.

**Lessons subsections:**

There has been little basic research on AGEP-related genetics, and the *CARD14* mutation may underlie several pustular rashes, including AGEP and generalized pustular psoriasis. Follow-up studies and further accumulation of patient data are required.

## 1. Introduction

Acute generalized exanthematous pustulosis (AGEP) is an acute-onset, nonfollicular, aseptic impetigo with a rash characterized by densely distributed pinhead-sized to soybean-sized pustules with diffuse flushing, usually associated with fever and leukocytosis.^[1]^ Its cause is not fully clear, and reports have indicated a relationship with drug sensitization, mainly involving anti-infective, antimalarial, and antihypertensive agents. In a few patients, AGEP is triggered by infection or other rare causes.^[2]^ AGEP occurs in approximately 1 to 5 per 1,000,000 patients per year, and approximately 20% have systemic involvement (mainly hepatic, renal, and pulmonary). Severe cases may cause multiple organ disorders, disseminated intravascular coagulation, and death.^[3]^

Hydroxychloroquine (HCQ) is an oral antimalarial drug with immunomodulatory effects that is commonly used for the long-term treatment of autoimmune diseases.^[4]^ HCQ is thought to be a rare cause of AGEP; however, the pathogenesis is unclear. AGEP is a T-lymphocyte-mediated disease associated with *IL36RN* mutations, suggesting a genetic basis for the pathogenesis of AGEP.^[5]^ However, a *CARD14* mutation with no clear link between AGEP and *CARD14* mutations was recently discovered. We report a case of AGEP associated with HCQ use and a newly discovered *CARD14* mutation in a patient with rheumatoid arthritis (RA).

## 2. Case presentation

The research was carried out following the principles stated in the Declaration of Helsinki and obtained the approval from the Ethics Committee at the Second Affiliated Hospital of Guizhou University of Traditional Chinese Medicine.

A 28-year-old woman with RA was admitted to the hospital with multiple pruritic rashes covering the body for 5 days. More than 2 months prior, she started leflunomide treatment for RA; however, she experienced no overt relief of joint pain. The medication was switched to HCQ 5 days prior to rash onset; thereafter, she experienced some pain relief. However, she developed erythema and a large number of pimples with severe pruritis that gradually spread to the head, face, trunk, and limbs. The symptoms seriously affected her daily life and sleep and did not improve significantly after using cetirizine. An intravenous infusion of dexamethasone and calcium gluconate administered at the local hospital was ineffective. She presented to our hospital for further treatment.

**Figure 1.**
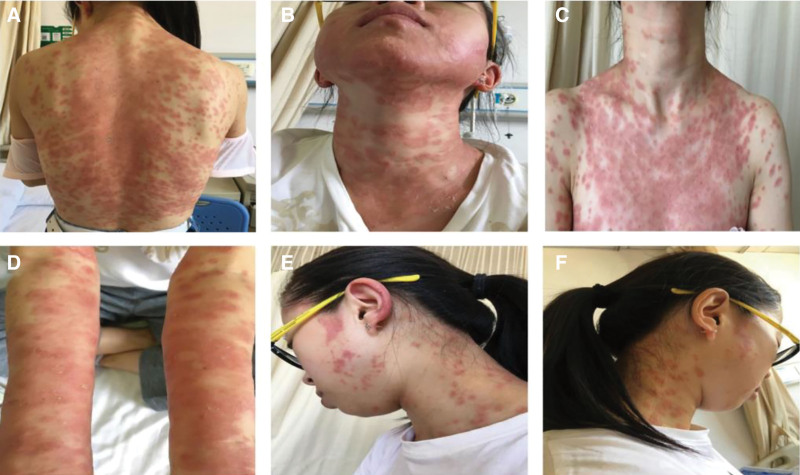
Patient’s clinical presentation. (A–D) Clinical findings at the first visit. (E and F) Skin rash after 15 days in hospital.

**Figure 2.**
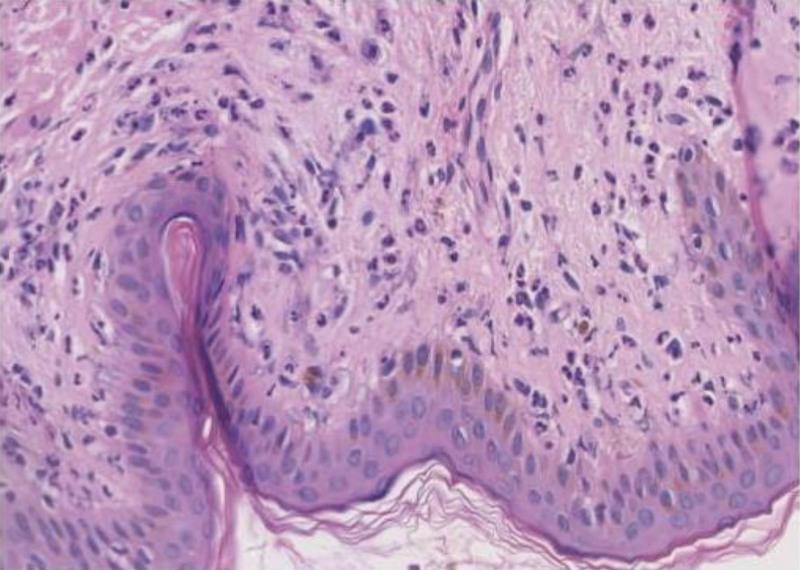
Histopathological examination of a skin biopsy specimen and mutation site detected in the samples. Back skin: epidermal hyperkeratosis, focal dyskeratosis, scattered neutrophil infiltration within the epidermis and superficial dermis, and spongiform pustules of the epidermis (hematoxylin & eosin, original magnification ×400).

A physical examination revealed stable vital signs. She had extensive edema and erythema on the trunk and extremities, with scattered or dense corn nib-sized erythematous papular and maculopapular lesions, with erosion and severe pruritis within the rash. No scratches, scabbing, scaling, or exudation were present (Fig. 1A–D). A blood evaluation revealed an elevated white blood cell count, neutrophil ratio, absolute neutrophil count, and C-reactive protein level. Histopathological examination revealed epidermal hyperkeratosis, focal keratosis, scattered neutrophil infiltration within the epidermis and superficial dermis, and epidermal cavernous pustules (Fig. 2A). To investigate genetic mutations, a venous blood sample was obtained for DNA analysis. A *CARD14* mutation was identified through whole-exome sequencing and Sanger verification (Table 1); however, no *IL36RN* mutations were identified. According to the patient’s symptoms, medical history, and histopathological examination results, as well as lesions suggestive of epidermal cavernous pustule formation, AGEP was diagnosed.

**Table 1:** Details of detected *CARD14* gene variant.

Gene	Chr	Nucleotide exchange	Amino acid exchange	Population genetics					
				1000G_ALL	1000G_EAS	ESP6500_ALL	ESP6500_AA	ExAC_ALL	ExAC_EAS
CARD14	Chr17	p.Glu211Glu	c.633G>A	0.347	0.2212	0.39	0.41	0.4547	0.305
CARD14	Chr17	p.Thr808Ala	c.2422A>G	0.3586	0.5129	0.41	0.26	0.4904	0.5426
CARD14	Chr17	p.Arg820Trp	c.2458C>T	0.353	0.499	0.41	0.25	0.4244	0.4728

1000G_ALL = 1000G mutation frequency in all populations, 1000G_EAS = mutation frequency in 1000G Asian populations, ExAC = Exome Aggregation Consortium, ESP6500_AA = ESP6500 mutation frequency in Black Americans, ESP6500_ALL = ESP6500 mutation frequency in all populations.

After admission, the patient was administered ketotifen fumarate tablets, dexamethasone sodium phosphate, calcium gluconate injection, methylprednisolone injection, vitamins C and B12, hydrocortisone butyrate cream, Reed acne cream, potassium chloride tablets, and pantoprazole capsules. After 15 days in the hospital, the rash improved, and the patient was discharged (Fig. 1E and F). After discharge, the rash gradually subsided, the original rash began crusting, and the aging epidermis desquamated. Fifteen days after discharge, all indicators were normal, and the rash had resolved.

## 3. Discussion

In our case, the patient had a clinical picture and histopathological findings that met the diagnostic criteria of AGEP.^[6]^ The illness had a clear temporal correlation with HCQ treatment. Moreover, after stopping the HCQ and receiving anti-allergic treatment, infection prevention, and symptomatic treatment, the rash gradually subsided.

AGEP is commonly regarded as a delayed hypersensitivity reaction mediated by lymphocytes. The production of interferon-γ, tumor necrosis factor-α, and granulocyte-macrophage colony-stimulating factor is stimulated by activated CD4+ T cells, thereby enhancing the formation of sterile pustules by neutrophils. This also promotes the release of chemokine 8 (interleukin [IL]-8), a potent neutrophil colony-stimulating factor that causes neutrophil aggregation, chemotaxis into vesicles, and the formation of sterile pustules in the blisters. Th17 cells play a role in the development of AGEP by producing IL-22 and IL-17, both of which stimulate keratinocytes to release IL-8.^[7]^

In genetic terms, *IL36RN* and *CARD14* mutations are associated with AGEP in some patients. IL-36 belongs to the -1 family of cytokines that activate intracellular regulators, such as pro-inflammatory nuclear factor kappa B and MAPK, by binding to specific IL-36R, thereby activating T cells, keratinocytes, and epithelial cells to cause skin inflammation.^[8]^ Nakai et al^[8]^ reported a case of dihydrocodeine-induced AGEP in a patient with an *IL36RN* c.28C>T heterozygous mutation. Navarini et al^[9]^ reported that a patient with amoxicillin-induced AGEP had an *IL36RN* c.338C>T homozygous mutation. CARD14 is a nuclear factor kappa B activator mainly expressed in the epidermis, resulting in activation of nuclear factor kappa B and MAPK signaling pathways, which, in turn, activate downstream signaling pathways to trigger inflammation.^[10]^ Podlipnik et al^[11]^ found that AGEP was associated with *CARD14* mutations and detected a heterozygous c.1288C c.1288C>T transition resulting in p.(Arg430Trp).

In our case, no *IL36RN* mutations were found; however, mutations have been detected in the CARD14 gene, with three mutation sites identified. Thus far, CARD14 mutations have been associated with several systemic pustular rash diseases, including generalized pustular psoriasis (GPP), palmoplantar psoriasis, psoriatic arthritis, and psoriasis. rash, erythema.^[12,13]^ Other CARD protein mutations have been reported to cause pustular skin diseases, such as Blau syndrome (*CARD15*/*NOD2* mutation).^[14]^

Lesions generally disappear 15 days after discontinuation of the medication causing AGEP because they are self-limiting and have a short course. The overarching principle of AGEP therapy comprises eliminating the suspected trigger and providing symptomatic supportive care while avoiding antibiotics whenever possible, except when there is a high suspicion of co-infection. Additionally, careful selection of antibiotics for patients with co-infection is important. Patients with mild disease can be treated with topical emollients. In contrast, Severely ill patients require systemic corticosteroids to relieve itching, suppress telangiectasia and inflammation, and shorten the course of the disease. For refractory disease, systemic therapy, including a combination of dapsone and cyclosporine, is recommended.^[1]^ In our case, the patient was administered systemic corticosteroids plus antihistamines, and the lesions completely resolved within 30 days.

GPP is mainly distinguished from HCQ-related AGEP by recurrent attacks, before the emergence of pustules, typical psoriasis lesions manifest as pustules on the initial plaque or rash. These pustules undergo expansion and fusion, resulting in the formation of “pus lakes.” Certain individuals exhibit a familial background of the ailment. Examining the histopathology showcases an abundance of psoriatic epidermal hyperplasia featuring keratosis. In the upper spinous layer, there can be visible Kogoj micro-abscesses, alongside potential instances of superficial dermal capillary dilation.^[15]^The patient did not have any prior occurrence or hereditary connection with psoriasis. Post HCQ treatment, the eruption manifested, with histopathological findings indicating the formation of pustules, aiding in the differentiation between AGEP and GPP. Therefore, histopathological examination is a vital method of clinically distinguishing AGEP from GPP in patients using HCQ. When encountering patients with suspected AGEP, there are conditions for which a skin biopsy is performed, and the pathological results help with the differential diagnosis of AGEP.

In conclusion, the *CARD14* mutation may underlie several pustular rashes, including AGEP and GPP. It is unclear what causes HCQ-induced AGEP with *CARD14* mutations, and a single case does not establish a definitive theory. Therefore, in order to enhance our comprehension of AGEP linked to CARD14 mutation, it is imperative to gather additional patient data encompassing information pertaining to the CARD14 mutation. Moreover, closely observing the clinical progression will be indispensable in this endeavor.

## Acknowledgments

We thank the hospital for administrative support, as well as BGI for technical support.

## Author contributions

**Conceptualization:** Wu-Kai Ma.

**Data curation:** Chang-Ming Chen.

**Software:** Xue-Mei Yuan, Hong Xiong.

**Writing – original draft:** Feng Luo.

**Writing – review & editing:** Xue-Ming Yao.
